# The poplar *VQ1* gene confers salt tolerance and pathogen resistance in transgenic *Arabidopsis* plants via changes in hormonal signaling

**DOI:** 10.1093/g3journal/jkac044

**Published:** 2022-02-23

**Authors:** Shifan Liu, Zhaocheng Wang, Jing Wu, Caijuan Wu, Rui Xiong, Yan Xiang, Hanwei Yan

**Affiliations:** 1 Laboratory of Modern Biotechnology, School of Forestry and Landscape Architecture, Anhui Agricultural University, Hefei 230036, China; 2 National Engineering Laboratory of Crop Stress Resistance Breeding, College of Life Sciences, Anhui Agricultural University, Hefei 230036, China

**Keywords:** salt, disease, *VQ1*, hormone

## Abstract

The VQ protein family is plant-specific, and is involved in growth, development, and biotic and abiotic stress responses. In this study, we found that the gene expression of poplar *VQ1*(Potri.001G029700) from *Populus trichocarpa* varied remarkably under salt stress and hormones associated with disease. A subcellular localization experiment showed that VQ1 was localized in the nucleus and cytomembrane in tobacco. The overexpression of *VQ1* in *Arabidopsis thaliana* enhanced its resistance to salt stress and disease, and was also responsive to it through abscisic acid. Compared with wild-type, transgenic *Arabidopsis* lines had significantly increased levels of abscisic acid and salicylic acid. The expression of some stress-related genes, such as *MPK6*, *NPR1*, and *PDF1.2*, was significantly up-regulated by salt in transgenic plants, while *WRKY70*, *ABI1*, *KUP6*, and *NCED2* were significantly down-regulated by *Pseudomonas syringae* pv. tomato DC3000 in transgenic plants. Together, these results demonstrate that *VQ1* modulates hormonal signaling to confer multiple biotic and abiotic stress responses in transgenic *Arabidopsis* plants.

## Introduction 

The environment has a crucial impact on plant growth and development (Zhu 2016), with several environmental factors having negative, and even harmful, effects on plants. These include biotic stresses, for example diseases, insect pests, and weeds, and abiotic stresses such as drought, salinity, and high and low temperatures. Salt stress is a major abiotic challenge in northern China where poplars are widely distributed (Song *et al.* 2016; Ke *et al.* 2017). Plant pathogens also present a major threat that constrains plant growth and development (Miller *et al.* 2009). In response to these environmental challenges, plants have evolved complex molecular mechanisms to sense and respond to these pressures by morphological and physiological variations (Zhu 2002; Bohnert *et al.* 2006; Chen *et al.* 2012).

Proteins containing VQ motifs, characterized by the conservative core sequence FxxhVQxhTG (where h denotes hydrophobic residues, and x indicates any amino acid), form a plant-specific protein family (Pecher *et al.* 2014). They have been reported to regulate plant growth, seed development, and stress responses (Jing and Lin 2015). The *AtVQ9* gene also has a role in negatively regulating salt tolerance of Arabidopsis, and by exploring its molecular mechanism, it was found that AtVQ9 acts as a transcriptional regulator, binding to the promoter region W-box of the downstream gene *RD29A* thus reducing Arabidopsis salt tolerance (Perruc *et al.* 2004; Hu *et al.* 2013b). Transgenic Arabidopsis overexpressing *AtVQ15* is highly sensitive to salt stress during the seed germination and seedling growth stages, however, the Arabidopsis *vq15* mutants showed a strong salt tolerance (Perruc *et al.* 2004; Hu *et al.* 2013b). While *PeVQ28* positively regulates the salt tolerance mediated by an abscisic acid (ABA)-dependent signaling pathway (Cheng *et al.* 2020).

Moreover, *Arabidopsis VQ23* actively regulates resistance to necrotizing and biotrophic pathogens (Lai *et al.* 2011), and *AtVQ4/MVQ1* over-expression in *Arabidopsis* reduces Flg22-induced resistance and negatively regulates pathogen-associated molecular pattern-induced resistance to pathogens (Pecher *et al.* 2014). Arabidopsis AtVQ22/JAV1 protein regulates the expression of defensively related gene *PDF1.2* on the Arabidopsis resistance to *B. cinerea* (Wang *et al.* 2010). AtVQ14 and AtWRKY10 interactions determine endosperm growth and seed size (Wang *et al.* 2010). OsMPK6-OsVQ13/OsWRKY45 signaling pathway influence rice grain size induced by JA (Wang *et al.* 2010). Knockdown *gmvq58* can strongly increase the expression of downstream defense-related genes *GmVSPβ* and *GmN: IFR*, to improve soybean resistance to common ground worm (Wang *et al.* 2010). Collectively, these results suggest that some of VQ proteins play important roles in plant growth and responses to environmental stresses.

Most functional studies of *VQ* genes have been conducted in *Arabidopsis*, and little is known about their role in trees. We previously identified 51 *VQ* members in poplar and carried out genome-wide analysis of the *VQ* gene family (Chu *et al.* 2016). In the present study, the molecular function of poplar *VQ1* was further studied under long-term salt stress and disease-related hormones. We transferred poplar *VQ1* into *Arabidopsis* and detected an increased tolerance to salt and pathogens in transgenic plants. Moreover, the hormone content of ABA and salicylic acid (SA) increased significantly in transgenic *Arabidopsis* compared with wild-type (WT). Our results indicate that poplar *VQ1* modulates differences in hormonal signaling to confer multiple biotic and abiotic stress responses.

## Materials and methods

### Plant materials, growth conditions, and treatments

The growth conditions for poplar seedlings have been described previously (Duan *et al.* 2015). For quantitative real-time (qRT)-PCR expression analysis, 3-week-old poplar seedlings were subjected to stress treatment, then underwent RNA extraction. *Pseudomonas syringae* pv. tomato DC3000 (Pst DC3000) was used to inoculate *Arabidopsis thaliana* seedlings as described previously (Jiang *et al.* 2016).

Seeds of tobacco (*Nicotiana tabacum* L.), WT *Arabidopsis* [Colombia (Col-0) ecotype], and 3 *Arabidopsis* T3 transgenic lines (L1, L17, and L21) were grown on MS agar medium at 4°C for 3 days to undergo vernalization, then transferred to a greenhouse [at 24°C, 16 h/8 h (light/dark), 80% relative humidity] for 10 days. Plants were then grown in a mixture of nutrient soil and vermiculite (3:1, v/v) at 22°C and 60% relative humidity with a 16-h/8-h day/night cycle.

### RNA-sequencing analysis of *VQ1* expression profiles and qRT-PCR

We retrieved RNA-sequencing (RNA-Seq) data of *VQ1* from the ArrayExpress archive of functional genomics data (accession number E-MTAB-5540) (Filichkin *et al.* 2018) and GSE109609 (Filichkin *et al.* 2018), which included data from plants exposed to salt and disease. The expression levels of *VQ1*, and genes associated with ABA and SA were investigated by qRT-PCR using primers designed with Primer Express 5.0 software (Supplementary Table 1).

### Isolation of *VQ1* and generation of Arabidopsis transgenic lines

The cDNA fragment of *VQ1* containing an open reading frame with a *Kpn* I restriction site at the 5′ end and a *Bam* HI site at the 3′ end was amplified by PCR using the following primers:

5′-GGGGTACCATGGATGTACTTGG-3′′ and 5′-CGGGATCCTTAATTAAGCACATCTAAC-3′′.

This coding sequence was then cloned into the vector pCAMBIA1301a (Rebuild and saved by our own laboratory) under the control of the CaMV35S promoter. The constructs were transformed into *Arabidopsis* Col-0 plants by *Agrobacterium*-mediated transformation as previously described (Wu *et al.* 2017). T1 and T2 transgenic plants were further confirmed by β-glucuronidase staining of seedlings and PCR analysis. Three generations of T3 *Arabidopsis* plants (Line 1, Line 17, and Line 21) were selected for further stress tolerance studies as described below.

### Subcellular localization analysis

The complete coding sequence of *VQ1* without a stop codon was inserted into the vector pCambia1305 (Abcam, Cambridge, MA, USA), which includes the CaMV 35S promoter and green fluorescence protein gene (*GFP*), using the following primers: forward 5′-GCTCTAGAATGGATGTACTTGGAGTTAAC-3′′ (containing an *Xba* I site) and reverse 5′-CGGGATCCATTAAGCACATCTAACTGAAAAG-3′′ (containing a *Bam* HI site). After obtaining a fusion plasmid of *VQ1* and the pCambia1305 vector, they were separately transformed into *Agrobacterium* *tumefaciens* GV3101. The suspension was injected into the leaves of *N. tabacum* plants, which were placed in a greenhouse at 25°C for 60 h in the dark. Then the fluorescence signal of GFP was observed by a LSM710 confocal microscope (Carl Zeiss, Jena, Germany) (Zhang *et al.* 2006). RFP is cell membrane marker gene as control. In the image of multilabel fluorescent sample, because 2 or more fluorophores are very close to each other in the microstructure, there will often be emission signal superposition, and this effect is called colocalization.

### Stress resistance of transgenic *Arabidopsis*

First, a seed germination experiment was performed. WT and transgenic *Arabidopsis* plants were cultured under identical conditions [24°C, 16 h/8 h (light/dark), and 80% relative humidity], and seeds were collected. These were germinated on plates containing MS medium supplemented with ABA (0, 0.6, 0.9, or 1.2 µM). The germination frequency was calculated after 3–5 days, and the main root length of seedlings was measured. Seeds were also germinated on standard MS medium, then seedlings transferred to MS plates containing ABA (0, 10, 30, or 50 µM) or NaCl (0, 100, 150, or 200 mM). After 7 days of growth, the main root length of seedlings were recorded.

Then, 3-week-old T3 transgenic lines (L1, L17, and L21) and WT *Arabidopsis* seedlings planted in the same pot were treated with salt solution (100, 200, or 275 mM NaCl), and control seedlings were irrigated with water. The pots were randomly arranged in a greenhouse and grown at 24°C with 16 h/8 h (light/dark), and 80% relative humidity. After 10–15 days, the pots were irrigated with water, and plants were allowed to recover for 7 days.

To determine the potential role of *VQ1* in disease resistance, transgenic and WT plants were inoculated with PstDC3000. After 3 days, the extent of leaf yellowing and wilting was observed (Jiang *et al.* 2016).

### Determinations of plant endogenous hormone levels and hormone-related genes’ expression

Endogenous hormone levels were determined in 3 strains of poplar *VQ1*-overexpressing *Arabidopsis* plants. The extraction and purification of ABA and SA were performed as previously described (Wang *et al.* 2009). Mass spectrometry [UHPLC-QE-MS（Agilent 1290 UHPLC-Thermo Q Exactive Focus/Plus/HF）] were performed, using 3 biological replicates for each set of experiments.

The expression of hormone-related marker genes was also analyzed by qRT-PCR.

## Results

### Expression pattern analysis of *VQ1*

**Fig. 1.**
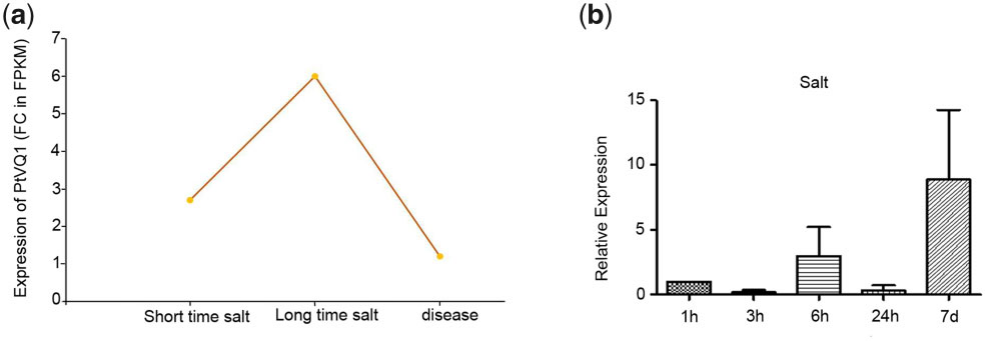
a) Showing expression dynamics of predicted *VQ1* genes. The y axis is the highest fold change(FC)of expression levels(in FPKM) during development. b) Expression pattern of VQ1 in poplar by qRT-PCR.

Abiotic and biotic stress can affect the growth and development of plants, and may induce the expression of stress-related genes. RNA-Seq data were used to analyze the expression of poplar *VQ* genes under salt stress and disease treatment. As shown in Fig. 1a, the expression of *VQ1* was significantly increased in poplar leaves under long-term salt stress and disease treatment.

The expression level of *VQ1* from *Populus davidiana×P. bolleana* was qualified by qRT-PCR (Fig. 1b), and the results shown to be consistent with *VQ1* RNA-Seq data.

### Subcellular localization of VQ1

**Fig. 2.**
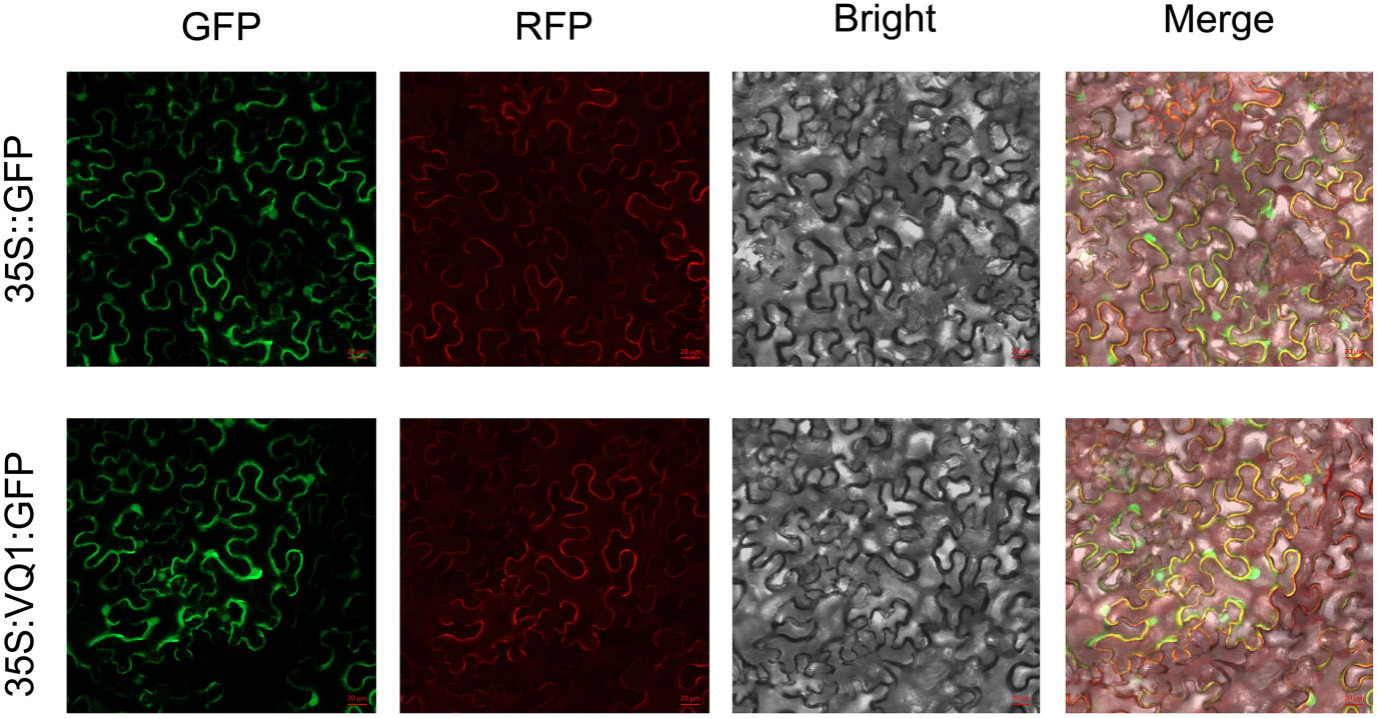
Nuclear localization of *VQ1and RFP*. The 35S::GFP::*VQ1* construct and the control vector 1305(35S::GFP) were transformed in *Nicotiana tabacum* leaves. Cell mem mar gene:: RFP as control.

To investigate the subcellular localization of VQ1, the full-length VQ1-GFP fusion vector without the stop codon and the GFP expression vector, both driven by the CaMV 35S promoter, were independently transformed into *N. benthamiana* leaf epidermal cells using *A. tumefaciens*. At the same time, we corotated a membrane marker and made colocalization. The results showed RFP and GFP signals can overlap. As a result, confocal laser microscopy analysis revealed that the VQ1-GFP fusion protein was expressed in the nucleus and cytomembrane, as was the control protein(Fig. 2).

### Resistance of *VQ1*-overexpressing Arabidopsis plants to salt stress and Pst DC3000

**Fig. 3:**
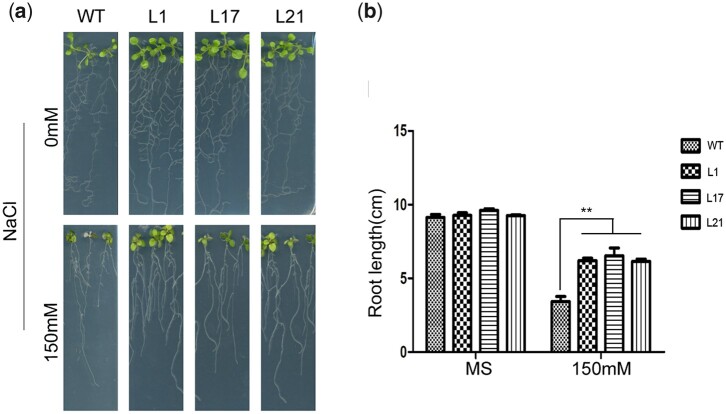
Response of *VQ1* transgenic *Arabidopsis thaliana* to NaCl. a) Germination performance of *VQ1* -overexpression and WT root length on MS medium containing 0, or 150mM NaCl. b) Calculation of the root length of transgenic and WT plants. Values are means±SE (*n* = 10). *P < 0.05, *t*-test; **P < 0.01, *t*-test.

Main root length assays were conducted on MS plates in the presence of 0 or 150 mM NaCl. No significant differences were observed among the growth rates of the 3 transgenic strains (L1, L17, and L21) and that of WT seedlings on the 0 mM NaCl MS plate. However, under 150 mM NaCl treatment, transgenic seedlings exhibited less severe growth inhibition than WT seedlings (Fig. 3a). The main root lengths of *VQ1-*overexpressing lines (3.9–4.1 cm) showed less inhibition than that of WT (2.1 cm) (Fig. 3b).

**Fig. 4.**
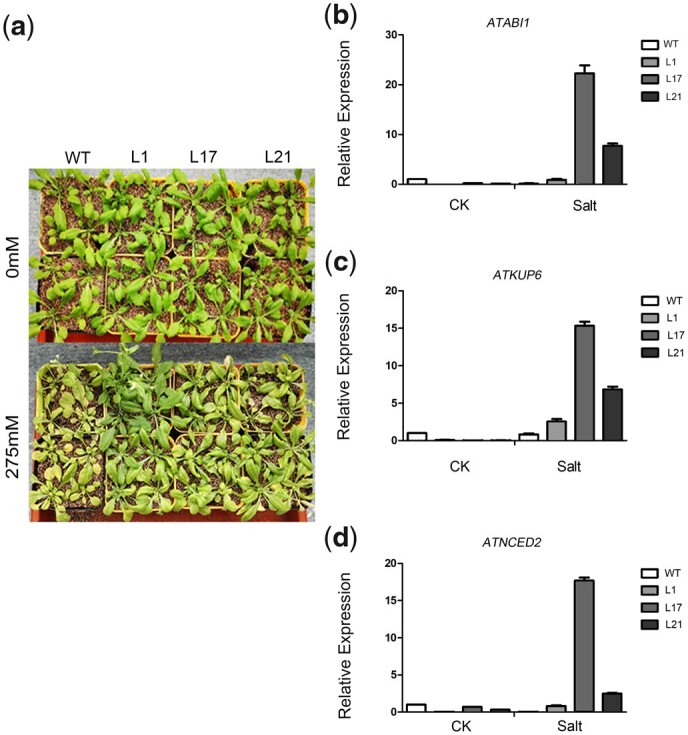
Salt stress of *VQ1* in transgenic Arabidopsis plants. a) Performance of WT and transgenic plants before and after salt treatment with 275mM NaCl. b–d) Relative expression levels of stress/ABA-responsive genes in transgenic Arabidopsis plants under normal salt stress. Leaves of WT and transgenic Arabidopsis plants were sampled after salt stress. *Y*-axis: relative expression levels; *x*-axis: different treatment conditions. Mean values±SE (*n* = 6).

**Fig. 5.**
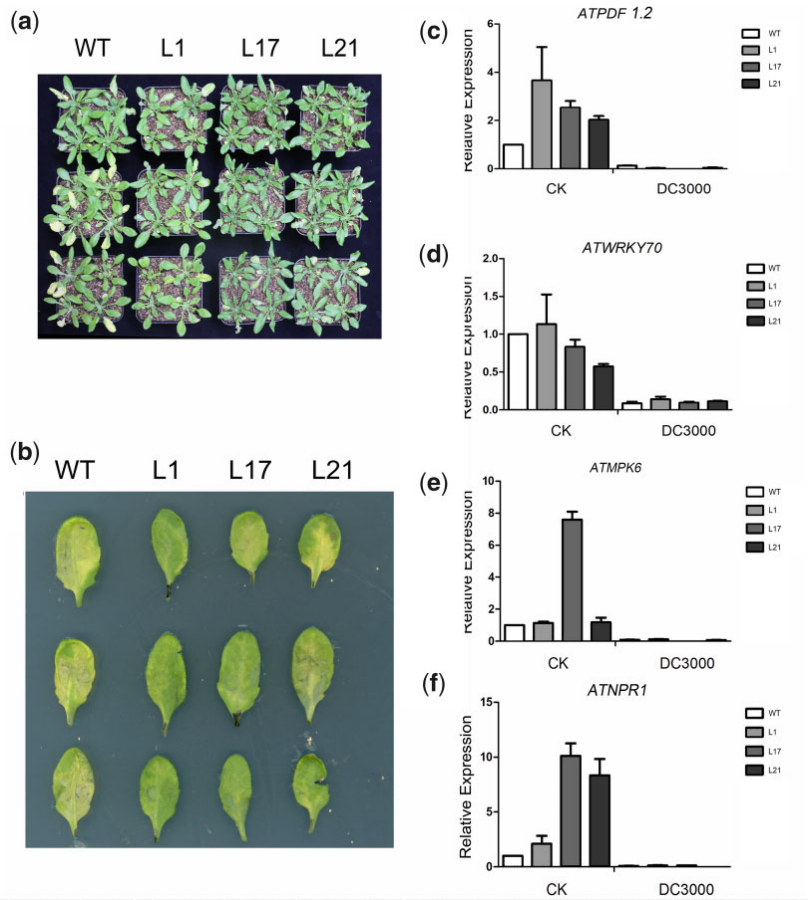
Abiotic stress of *VQ1* in transgenic Arabidopsis plants. a,b) Performance of WT and transgenic plants before and after salt treatment with DC3000. c–f) Relative expression levels of stress/SA and MeJA responsive genes in transgenic Arabidopsis plants under normal salt stress. Leaves of WT and transgenic Arabidopsis plants were sampled after salt stress. *Y*-axis: relative expression levels; *x*-axis: different treatment conditions. Mean values±SE (*n*=6).

On the basis of this phenotype assessment, we analyzed the growth and selected stress-related parameters of WT and transgenic plants in response to treatment with 275 mM NaCl. After 15 days of treatment, most WT plants had withered, whereas the transgenic lines grew well (Fig. 4a). Similarly, 3 days after the injection of DC3000 with an optical density of 0.002, WT leaves were more prone to wilting than those of transgenic plants. The extent of WT leaf yellowing was much greater than that of transgenic plants (Fig. 5, a and b).

### *VQ1*-overexpressing Arabidopsis plants in response to ABA

As an important component of plant signaling pathways, ABA acts as a key signal for regulating a range of plant physiological processes such as germination, seedling growth, root development, stomatal regulation, and defense to osmotic stress (Cutler *et al.* 2010; Clouse 2016). Because several VQ proteins are responsive to ABA stress, we next investigated the role of *VQ1* in the ABA signaling pathway. We also evaluated whether *VQ1* affected ABA signaling by examining the ABA sensitivity of *VQ1*-transgenic plants in a seed germination experiment.

**Fig. 6.**
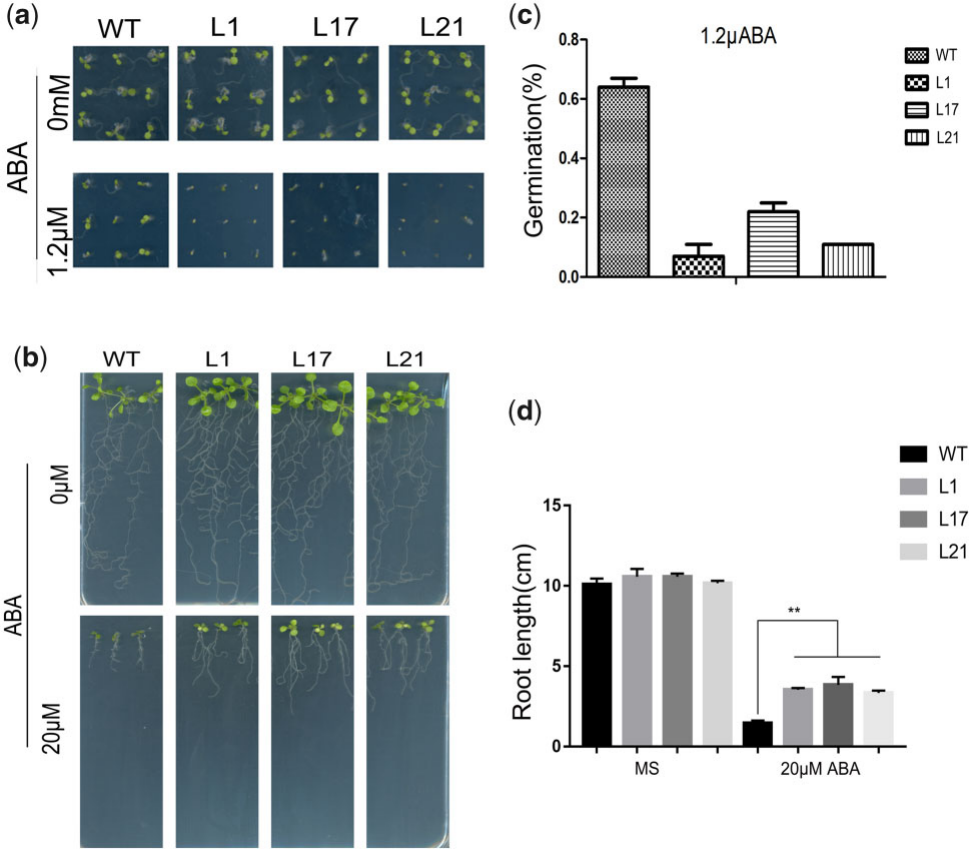
Response of *VQ1* transgenic *Arabidopsis thaliana* to ABA. a) Germination performance of *VQ1*-overexpression and WT seeds on MS medium containing 0, or 1.2 μM ABA. b) Effect of salt stress on root length of transgenic and WT plants. c) Calculation of the germination rates of transgenic and WT seeds. d) Calculation of the root length of transgenic and WT plants. Values are means±SE (*n* = 10). *P < 0.05, *t*-test; **P < 0.01, *t*-test.

Under control conditions, *VQ1*-overexpressing transgenic plants showed no difference in germination frequency from WT. However, in response to supplementation with 1.2 µM ABA, the germination frequency of WT seeds decreased; the germination frequency of transgenic seeds also decreased but to a lesser extent than that of WT seeds (Fig. 6, a and c). Meanwhile, the root growth of WT and *VQ1*-overexpressing seedlings was determined under treatment with 0 or 20 µM ABA. No significant difference was observed in the main root length between WT and *VQ1*-overexpressing seedlings under control conditions; however, in response to 20 µM ABA treatment, the growth of *VQ1*-transgenic seedlings was restrained less severely than that of WT seedlings (Fig. 6, b and d). Therefore, poplar *VQ1* overexpression increased the response of *Arabidopsis* seedlings to ABA.

### Changes in the endogenous hormone concentrations

**Fig. 7.**
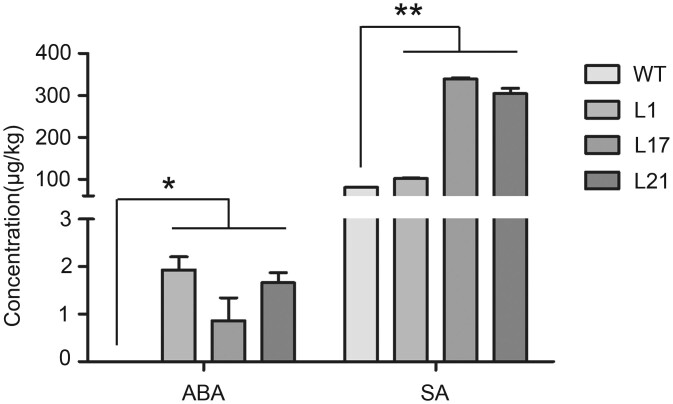
In 3 *Arabidopsis thaliana* lines overexpressing *VQ1* and WT lines, dynamic changes in the concentrations (μg kg^−1^ tissue) of 2 endogenous hormones. The 3 means ± SE were analyzed using an ANOVA (*P < 0.05 and **P < 0.01).

To better understand the key roles of the plant hormone signaling pathway in regulating the growth and development of *VQ1*-overexpressing *Arabidopsis* plants, we quantified the concentrations of endogenous hormones ABA and SA using Mass spectrometry [UHPLC-QE-MS（Agilent 1290 UHPLC-Thermo Q Exactive Focus/Plus/HF）] (Fig. 7). The levels of ABA, and SA, in the 3 transgenic lines were significantly increased compared with WT levels.

### Expression of ABA-related and SA-related genes in *VQ1*-overexpressing *Arabidopsis* plants

To investigate the molecular mechanism of the *VQ1*-mediated response to stress conditions, we next analyzed the expression levels of 7 marker genes (*AtKUP6*, *AtNCED2*, *AtABI1*, *AtWRKY70*, *AtNPR1*, *AtPDF1.2*, and *AtMPK6*) in *VQ1*-overexpressing *Arabidopsis* lines. In response to 5 days of salt treatment, *AtKUP6*, *AtNCED2*, and *AtABI1* showed higher expression levels in transgenic plants than in WT plants (Fig. 4, b–d). In response to 3 days of Pst DC3000 treatment, *AtWRKY70*, *AtNPR1*, *AtPDF1.2*, and *AtMPK6* showed lower expression levels in transgenic plants than in WT plants (Fig. 5, c–f).

## Discussion

Previous genome-wide bioinformatics and functional analyses of *VQ* genes have mainly been conducted in *Arabidopsis*, and included the analysis of *AtVQ9* (*AtMVQ10*) and *AtVQ15* (*AtCAMBP25*) (Perruc *et al.* 2004; Hu *et al.* 2013b). Our earlier work identified related genes in poplar, and studied their characteristics and evolution. However, no functional analyses have been reported for poplar *VQ* genes. In the present study, we used RNA-seq data to report a significant increase in the expression of *VQ1*, *VQ4*, *VQ11*, *VQ13*, *VQ19*, *VQ23*, *VQ34*, and *VQ45* in poplar leaves under long-term salt stress. Based on our current expression profiles combined with information about functional *VQ* genes reported in the literature, we selected the potential abiotic stress-related gene *VQ1* for functional analysis in *Arabidopsis*.

The over-expression of *VQ1* induces the expression of stress-responsive genes, which in turn leads to increased resistance to a diverse range of stresses. *AtABI1* encodes protein phosphatase 2C, which plays a major role in ABA-mediated signaling associated with stress responses. Under nonstress conditions, *AtABI1* showed similar expression levels in *VQ1*-overexpressing and WT plants. However, under salt stress, *AtABI1* expression was higher in transgenic plants than WT plants. *AtKUP6* and *AtNCED2* expression showed the same trend, suggesting that *VQ1* functions in stress-induced ABA biosynthesis. Therefore, the enhanced expression of these genes may enhance the salt stress tolerance of *VQ1*.

As a marker gene in the SA signaling pathway, *AtNPR1* expression was dramatically decreased in *VQ1-*overexpressing *Arabidopsis*. *AtWRKY70* was previously shown to activate the SA signal pathway (Li *et al.*^2004^, ^2006^), and its expression in our study was lower in transgenic than WT plants under salt stress. These results indicate that the overexpression of *VQ1* reduces *AtNPR1* and *AtWRKY70* expression through antagonistic interactions via the SA signaling pathway.

Mitogen-activated protein kinase (MAPK) cascades are universal signaling modules in eukaryotes that function in multiple pathways. *MPK3/MPK6* and their orthologs from various plant species are associated with positively regulating pathogen defense (Andreasson and Ellis 2010). As a marker gene associated with SA, *AtPDF1.2* expression was dramatically decreased in plants overexpressing *VQ1*. This suggests that *VQ1* overexpression induces ABA synthesis in transgenic plants under salt stress, leading to increased signaling and ABA-related gene expression. *VQ1* overexpression also appears to induce the synthesis of SA in transgenic plants under biological stress, resulting in the decreased expression levels of SA-related genes. Thus, the increased resistance of *VQ1*-overexpressing plants to salt stress and other abiotic stresses may result, in part, from the enhanced expression of these genes.

Protein–protein interactions are the basis for all cellular metabolic activities (Hu *et al.* 2013a). As a cofactor of transcription regulation, VQ protein can interact with a variety of transcription factors, affecting the ability of the interacting transcription factors to bind to target genes, and jointly regulate the growth and development of plants and respond to biological and abiotic stress factors (Hu *et al.* 2013a). Based on the phylogenetic tree, *VQ1* belongs to the same group as Arabidopsis *AtVQ1, AtVQ16*, and *AtVQ23* in Chu W ’s paper [33]. In previous studies, AtVQ23 was shown to interact with AtWRKY33 to increase plant disease resistance (Lai *et al.* 2011). AtVQ9 functions as a transcriptional repressor that antagonizes WRKY8 and negatively regulates salt tolerance [11]. The interaction of PeVQ28 and PeWRKY83 positively regulates the salt tolerance mediated by an abscisic acid (ABA)-dependent signaling pathway [12]. AtVQ4 is phosphorylated by MPK3/6, which weakens the inhibition of WRKY-mediated disease resistance signaling pathway and enhances the expression of downstream disease resistance genes [8]. while AtVQ14 interacts with AtWRKY10 to form a complex protein that affects seed size (Wang *et al.* 2010). The VQ-assisted WRKY transcription factor is involved in the regulation of plant growth and development, and response to abiotic and biotic stresses, and provided a pointer for exploration of the function of *VQ1*.

Finally, the root length of seedlings overexpressing *VQ1* was significantly higher than that of WT under ABA and salt treatment. Thus, *VQ1-*overexpressing plants show stronger salt stress resistance than WT. Furthermore, the 3 transgenic lines had significantly increased endogenous hormone levels, and altered expression of hormone marker genes compared with WT. Therefore, *VQ1* participation in salt tolerance and pathogen resistance appears to involve the actions of several hormonal pathways.

## Data availability

The genome sequences of Poplar was downloaded from the Poplar Database (https://phytozome-next.jgi.doe.gov/info/Ptrichocarpa_v4_1). The genome sequences of Arabidopsis was downloaded from the Arabidopsis Information Resource (https://www.arabidopsis.org).

The poplar RNA-seq data are downloaded from (https://www.ncbi.nlm.nih.gov/). We retrieved RNA-Seq data of VQ1 from the ArrayExpress archive of functional genomics data (accession number E-MTAB-5540) and GSE109609, which included data from plants exposed to salt and disease.

Supplemental material is available at *G3* online.

## Supplementary Material

jkac044_Supplementary_Data
